# P2Y1 Receptor as a Catalyst of Brain Neurodegeneration

**DOI:** 10.3390/neurosci3040043

**Published:** 2022-10-31

**Authors:** Ricardo J. Rodrigues, Ana S. Figueira, Joana M. Marques

**Affiliations:** 1 CNC—Center for Neuroscience and Cell Biology, University of Coimbra, 3004-504 Coimbra, Portugal; 2 Institute of Interdisciplinary Research, University of Coimbra, 3030-789 Coimbra, Portugal

**Keywords:** P2Y1 receptor, neurodegeneration, ATP, ADP, brain

## Abstract

Different brain disorders display distinctive etiologies and pathogenic mechanisms. However, they also share pathogenic events. One event systematically occurring in different brain disorders, both acute and chronic, is the increase of the extracellular ATP levels. Accordingly, several P2 (ATP/ADP) and P1 (adenosine) receptors, as well as the ectoenzymes involved in the extracellular catabolism of ATP, have been associated to different brain pathologies, either with a neuroprotective or neurodegenerative action. The P2Y1 receptor (P2Y1R) is one of the purinergic receptors associated to different brain diseases. It has a widespread regional, cellular, and subcellular distribution in the brain, it is capable of modulating synaptic function and neuronal activity, and it is particularly important in the control of astrocytic activity and in astrocyte–neuron communication. In diverse brain pathologies, there is growing evidence of a noxious gain-of-function of P2Y1R favoring neurodegeneration by promoting astrocyte hyperactivity, entraining Ca^2+^-waves, and inducing the release of glutamate by directly or indirectly recruiting microglia and/or by increasing the susceptibility of neurons to damage. Here, we review the current evidence on the involvement of P2Y1R in different acute and chronic neurodegenerative brain disorders and the underlying mechanisms.

## 1. Introduction

Different brain disorders display characteristic etiologies and phenotypes, yet they rely on common pathogenic events. One such common event in the development of neurodegeneration is excitotoxicity [1,2] observed both in acute and chronic brain diseases [3,4,5,6]. It involves an abnormal Ca^2+^-influx, mainly mediated by the high Ca^2+^-permeable NMDA receptors (NMDARs) [7,8], which then leads to the activation of calpains and other proteases mediating cytoskeleton damage [9] paralleled by oxidative stress, mitochondrial dysfunction, and subsequent activation of apoptotic pathways, events which are also associated with different acute and chronic neurodegenerative conditions [10,11,12,13].

Another event systematically occurring in diverse brain disorders is the increase of the extracellular levels of ATP. ATP is a ubiquitous intracellular metabolite in the millimolar range. Hence, any insult leading to damaged cell membranes of injured or dying cells causes a rapid increase in their extracellular levels. Accordingly, in mechanical injuries such as traumatic brain injury (TBI), there is evidence of increased ATP release [14,15,16,17]. This increase occurs not only proximal to the point of impact [16], but also in remote regions distant to the location of impact [15,17]. This indicates that the increase in extracellular ATP in TBI should reflect not only ATP leakage from injured cells, but also the involvement of mechanisms to release ATP, most likely from astrocytes [15,17,18]. Indeed, besides ATP leakage from dying cells, there are physiological mechanisms designed to active-release ATP, which are also observed in pathophysiological phenomena. ATP can be released or co-released from nerve terminals [19] in an activity and Ca^2+^-dependent exocytotic vesicular manner [20]. This occurs particularly at high-frequency stimulations [21,22,23], which suggests that this neuronal activity-dependent release of ATP may be contributing to seizure-induced ATP release [24,25]. Recently, we showed an increase in the evoked release of ATP from rat hippocampal nerve terminals 7 days upon the induction of status epilepticus (SE) [26]. In metabolic stress such as hypoxia or ischemia, the observed increase in ATP release [27,28,29] also seems to have a neuronal source [30]. Moreover, recently, it was shown that the *i.c.v.* injection of Aβ_1–42_, which is associated to Alzheimer’s disease (AD), increases the evoked release of ATP from mice hippocampal nerve terminals, as well [31]. Extracellular ATP may also arise from astrocytes, perhaps the major source of extracellular ATP, through a Ca^2+^-dependent vesicular release by exocytosis [32,33,34], by lysosomes [35], or through hemichannels formed by either pannexins [36,37,38] or connexins [39,40]. Aβ exposure promotes the release of ATP by astrocytes [41,42,43] through hemichannels, in particular, connexin-43 [41,43]. In a mouse model of familial AD, astrocytic hyperactivity was shown to be mediated by paracrine purinergic signaling involving connexin channels [44]. Pannexins have been also reported to be involved in neurotoxicity, namely, in ischemia [45], and has been proposed as a target for neuroprotection [46]. Accordingly, the increase of extracellular ATP during high-potassium-induced ictal discharges on slices obtained from resected tissues of TLE patients was blocked by the inhibition of pannexin-1, which also provided anticonvulsive effects in a mouse model of kainic acid (KA)-induced seizures [47]. This may involve astrocytic pannexin-1 [48]. Notably, neuronal pannexin-1 has also been shown to be activated during ischemia and may constitute an additional neuronal source of pathological extracellular ATP [49]. ATP release from astrocytes can also be mediated by maxi-anion channels as observed in oxygen–glucose deprivation [50]. Extracellular ATP may further arise through P2X7R, either directly through the high-permeability pore formed by prolonged P2X7R activation [51,52] or by a synergistic interaction with pannexins [53,54], suggesting a self-mechanism of sustained increase in the extracellular levels of ATP. In addition to neurons and astrocytes, microglia may also contribute to the release of ATP in pathological conditions, eventually through vesicular exocytosis [55]. For instance, in mouse primary microglial cells, fibrillar and oligomeric Aβ_1–42_ cause ATP release [56]; and in microglial N13 cells, the active Aβ_25–35_ peptide causes a dose-dependent release of ATP [57].

Thus, there is now compelling evidence demonstrating a sustained efflux of ATP into the extracellular milieu in brain disorders either through the leak of ATP through damaged membranes, or through mechanisms designed to active-release ATP in pathological phenomena. This supports the concept that extracellular ATP is a danger signal in the brain [58,59], constituting another event shared by different acute and brain disorders. Accordingly, several P2 receptors (P2R) and P1 receptors (P1R) were activated by adenosine upon the extracellular catabolism of ATP, and the respective ectoenzymes have been associated to different brain pathologies, either with neuroprotective or neurodegenerative actions (for review, see e.g., [59,60,61,62,63,64,65,66]). Here, we will review in particular the current knowledge on the contribution of the P2Y1 receptor (P2Y1R) to neurodegeneration in different acute and brain disorders and discuss the underlying mechanisms. 

## 2. The Multimodal P2Y1 Receptor

P2Y1R is a metabotropic receptor activated by ATP/ADP with a widespread regional cellular and subcellular distribution in the brain. In neurons, P2Y1R is located both pre- and postsynaptically [67] and non-synaptically both in dendrites, cell bodies, and axons [68]. Presynaptic P2Y1R modulates neurotransmitter release such as glutamate [67,69] or GABA [70], eventually through its ability to regulate N-type Ca^2+^-channels in neurons [71,72]. Postsynaptically, P2Y1R inhibits NMDAR [73], impacting the synaptic plasticity [74]. This may also rely on the inhibition of voltage-gated Ca^2+^- channels [75]. Recently, we have shown in cultured rat hippocampal neurons that P2Y1R modulates the NMDA-induced Ca^2+^-entry in a bidirectional and subcellular-specific manner, decreasing it in the soma and dendrites and increasing it in the axons, most likely reflecting a differential regulation of NMDARs’ density in the different cellular compartments [68]. P2Y1R also regulates GABA transmission by postsynaptic regulation of GABA_A_ receptors [76] and through the direct control of interneurons’ excitability in different regions [76,77,78,79,80], involving the regulation of K^+^-conductance [77,79,80]. P2Y1R can also promote rat hippocampal pyramidal neurons’ excitability through the inhibition of M-type K^+^-currents [81]. In astrocytes, P2Y1Rs are also highly expressed [44,82,83,84,85], playing a key role in the propagation of calcium waves throughout the astrocytic network [34,86,87]. P2Y1R also regulates Ca^2+^-dependent vesicular glutamate release from astrocytes [88], which is able to activate NMDAR on neurons [83] and modulate neuronal function [80,83,89,90]. It also regulates the release of ATP from astrocytes [91], sustaining an autocrine ATPergic signaling [90] and modulating synaptic function [92]. P2Y1R also controls GABA uptake from cultured rat cortical astrocytes [93]. Hence, P2Y1R regulates astrocytic function, modulating astrocytic network activity and gliotransmission. The expression of P2Y1R has been also reported in microglia [94,95,96]. While motility seems to be associated with P2Y12R [97,98], microglia process retraction may involve P2Y1R [99]. Altogether, the multiple functions so far ascribed to P2Y1R set a transcellular integrative role for P2Y1R in the brain.

## 3. P2Y1 Receptor in Neurodegenerative Disorders

An increase in the expression levels of P2Y1R has been documented in different acute or chronic neurological disorders such as epilepsy [100,101,102], mechanical injury [103], ischemia [84], or AD [44,104], which suggests the gain of a noxious function of P2Y1R. Accordingly, compelling evidence have been associating P2Y1R with different acute and chronic brain disorders. 

In ischemic conditions such as oxygen–glucose deprivation (OGD), the blockade of P2Y1R prevented the depression of field excitatory postsynaptic potentials and anoxic depolarization in rat hippocampal slices, also preventing CA1 pyramidal neuronal damage [105,106]. Similar neuroprotection was afforded by the *i.c.v.* administration of a selective antagonist of P2Y1R after transient middle cerebral artery occlusion in rats, reducing infarct volume and recovering motor coordination [84]. Moreover, P2Y1R-KO mice displayed reduced hippocampal damage and no cognitive decline upon middle cerebral artery occlusion, an effect mimicked by the pharmacological blockade of P2Y1R in rodents [107]. This has been associated to the control of astrocytic function and glial neuroinflammatory response [84,107,108]. However, neuronal mechanisms should also be involved in the deleterious contribution of P2Y1R in ischemic conditions since in another study, it was observed that P2Y1R blockade attenuated neuronal damage and cognitive performance induced by permanent middle cerebral artery occlusion, without inhibiting the astrocytic or microglial reactivity [109]. On the other hand, a neuroprotective action of P2Y1R has been also reported in ischemia. P2Y1R-KO mice displayed a higher number of injured hippocampal neurons upon OGD [110] and in mouse ischemic models of photo-thrombolysis, a reduction of neuronal damage was observed with the activation of astrocytic P2Y1R [111,112]. A similar neuroprotection provided by astrocytic P2Y1R was observed in oxidative stress through IL-6 release [113]. A neuroprotective vs. neurodegenerative action of P2Y1R may be due to either the degree of P2Y1R-driven activity and/or a time-dependent gain of a noxious function of P2Y1R, shifting astrocytes from a supportive role to a deleterious impact and/or a time-dependent differential impact of neuronal and glial P2Y1R. A similar time-dependent shift from a neuroprotective to a neurodegenerative input of P2Y1R was observed in excitotoxicity. P2Y1R was shown to be required for glutamate-induced synaptic loss and subsequent neuronal death in the rat hippocampus both in vitro and in vivo [68]. This is due at least in part to a P2Y1R-driven increase of NMDARs at the axon, leading to a deleterious Ca^2+^-entry and subsequent calpain-mediated axonal cytoskeleton damage [68]. However, it also provided evidence that P2Y1R may reduce AMPAR, decreasing the susceptibility of neurons to excitotoxicity [114]. In SE-induced neurodegeneration, the *i.c.v.* injection of a selective antagonist of P2Y1R reduced hippocampal neuronal death observed with the systemic *i.p.* administration of KA [68]. However, in a more recent study, it was detailed that there is a time-dependent shift from a neuroprotective to a neurodegenerative contribution of P2Y1R to SE-induced neurodegeneration, correlated with a different impact in SE-induced seizure activity. Using intra-amygdala KA and pilocarpine mouse models, while the antagonism of P2Y1R before SE induction increased seizure activity and neurodegeneration in the hippocampus, the blockade of P2Y1R shortly after the onset of SE reduced seizure activity and degeneration [115]. It was suggested that this may be due to a time-dependent contribution of neuronal and astrocytic P2Y1R [115]. Neuronal P2Y1R can reduce hyperexcitability, either by directly depressing postsynaptic NMDARs [68,73] and/or by a circuit-driven increase of the inhibitory tonus [77,78]; however, then the recruitment of astrocytes and the P2Y1R-induced release of glutamate [88], subsequently activating NMDAR on neurons [83], can lead to hyperexcitability [66,90,116,117]. In addition, this time-dependent neuroprotective to neurodegenerative shift may also be due to the fact that the contribution of neuronal and astrocytic P2Y1Rs may also change at different pathogenic stages. For instance, neuronal P2Y1R tonically depresses dendritic NMDARs, but in excitotoxic conditions, it induces a toxic increase in axonal NMDARs [68]. Interestingly, a similar P2Y1R-driven increase in NMDARs was found in the dorsal root ganglion underlying remifentanil-induced postoperative hyperalgesia [118]. However, this contribution of neuronal P2Y1R to neurodegeneration fades with more intense excitotoxic conditions [68]. Regarding the contribution of astrocytic P2Y1R, astrocytes have a physiological supportive role to neuronal function, namely, glutamate uptake or the release of neurotrophic factors [119] and, as mentioned, astrocytic P2Y1R can have a neuroprotective effect as observed in ischemia, oxidative stress [111,112,113], and TBI (see below) [120]. Nevertheless, the evidence so far provided essentially point to a net neurodegenerative contribution of P2Y1R in excitotoxic conditions. There is an increased density of P2Y1R upon SE as well as in human tissue from temporal lobe epilepsy patients [101,102], supporting microglia and astrocytic-induced hyperexcitability through the P2Y1R-induced release of glutamate from astrocytes [102,116,121]. This is further heralded by the observation that the blockade of P2Y1R post-SE delayed the onset of epilepsy and suppressed epileptic seizures in a reversible manner [115]. In addition to a control of seizure severity, the antagonism of P2Y1R may be also beneficial against epilepsy comorbidities since the blockade of P2Y1R rescued synaptic plasticity, associated to a normalization of astroglial Ca^2+^-activity in epileptic hippocampus [121]

The blockade of P2Y1Rs also afforded neuroprotection upon TBI even in remote regions from the injury site, improving cognitive outcomes [15]. This effect was dependent on P2Y1R-mediated astrocytic Ca^2+^-waves and on NMDAR activation [15], indicating an exacerbation/propagation of neuronal injury through a P2Y1R-driven release of glutamate from astrocytes. This is further sustained by the release of ATP in regions distant to the impact point [17]. In addition to having control of astrocytes, it was more recently shown that the blockade of P2Y1R suppressed microglial activation at the injury site [122]. Moreover, evidence was provided that microglia recruited to the injury core is important for the formation of neuroprotective astrocyte scar in the peri-injured region by downregulating P2Y1R in astrocytes [120]. Hence, the neuroprotection afforded by the inhibition of P2Y1R in TBI may be due by the concomitant promotion of a protective scar around the lesion, mimicking the beneficial effects of microglia but inhibiting the microglia-mediated inflammatory response and avoiding the astrocytic-driven hyperexcitability involved in the exacerbation and propagation of neuronal injury.

In AD, P2Y1Rs were found to colocalize with neurofibrillary tangles and amyloid β (Aβ) plaques characteristic to AD [104]. In an APP/PS1 AD mouse model, reactive astrocytes near Aβ plaques showed enhanced P2Y1R-mediated Ca^2+^ signaling, displaying both significantly higher resting Ca^2+^ levels and increased propagation of intercellular Ca^2+^-waves [44], and was suggested to mediate Aβ-induced synaptic dysfunction/loss and neuronal damage [44,123]. Indeed, more recently, it was shown that the chronic blockade of P2Y1R in the APP/PS1 mice reduced/normalized neuronal activity, restored synaptic plasticity and synaptic integrity, reduced neuritic dystrophy, and attenuated cognitive decline [124]. The observation that this was partly observed in mice lacking the IP3 receptor type 2, the signaling downstream of P2Y1R activation, indicates that it is in part due to the inhibition of the astrocytic hyperactivity, similar to that observed in epileptic hippocampus [121]. However, it also indicates that P2Y1Rs other than those located in astrocytes could also be involved. In fact, neuronal P2Y1R may also contribute to the initial synaptic dysfunction/loss by favoring the loss of axonal integrity, observed prior to dendritic damage and later neuronal death through an increase in NMDARs [68]. Besides, additional mechanisms may underlie the contribution of P2Y1R to AD-associated synaptic dysfunction, plasticity deficits, and cognitive impairment, eventually abnormally activated by astrocytic-derived ATP [80,92]. The depression of postsynaptic NMDARs [73,74,75] was shown to have an impact in synaptic plasticity, particularly in pathological conditions such as hypoxia [75]. The selective activation of P2Y1R in the medial prefrontal cortex was shown to be sufficient to impair working memory and learning [125]. In addition, the recently shown Aβ-associated disruption of inhibitory homeostasis mediated by P2Y1R [126] may also promote circuit-driven synaptic dysfunction. 

In summary, there is now compelling evidence associating P2Y1R to different acute and chronic neurodegenerative disorders with clear distinctive etiologies and pathogenesis, essentially pointing towards a pro-neurodegenerative action.

## 4. P2Y1 Receptor as a Catalyst of Neurodegeneration

**Figure 1:**
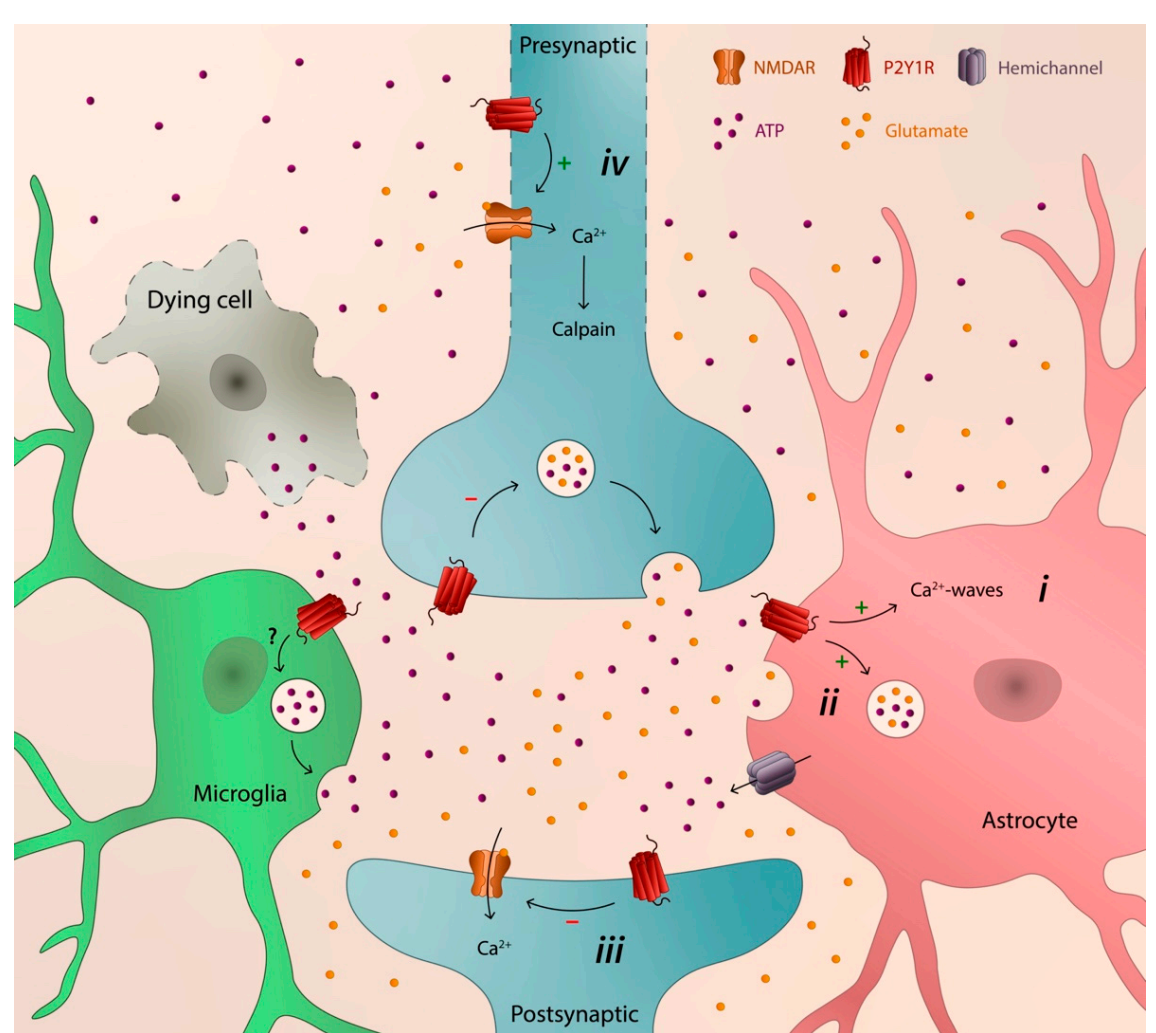
Schematic illustration depicting the transcellular capability of P2Y1R to catalyze neurodegeneration: (*i*) astrocytic hyperactivity; (*ii*) release of glutamate from astrocytes; (*iii*) depression of synaptic activity; and (*iv*) early axonal degeneration, synaptic loss, and later neuronal death.

The major mechanism by which P2Y1R favors neurodegeneration, shared by different brain disorders, is its ability to control astrocytic function, thus entraining Ca^2+^-waves, inducing the release of inflammatory cytokines [84], and promoting the release of glutamate [15,88,102,116,121], ultimately leading to hyperexcitability and neuronal damage [66,83,90,116,117]. P2Y1R inhibition is also neuroprotective by allowing the development of neuroprotective astrocytic scars, namely in TBI [120]. These deleterious mechanisms of astrocytic P2Y1R are further sustained/enhanced by P2Y1R itself due to its ability to prevent astrocytic damage upon different noxious insults [63,127,128,129] and by mediating the autocrine signaling, inducing a sustained release of ATP from astrocytes [17,44,91,116,130]. This mechanism can be also sustained or potentiated by microglia recruitment through the release of ATP and subsequent P2Y1R-driven stimulation of astrocytes, promoting glutamatergic gliotransmission with an impact in synaptic activity [85], tethering inflammation to synaptic failure. Besides, although the role of P2Y1R in microglia remains elusive, it has been shown that, either directly or indirectly, P2Y1R is involved in the recruitment of microglia in epileptic phenomena [102] in TBI [122] and in ischemia [107]. In addition, neuronal P2Y1R may also contribute to neurodegeneration [68,109], namely, by favoring the initial synaptic loss and later neuronal death by a subcellular-specific upregulation of NMDARs, increasing their density in axons, leading to an initial Ca^2+^-driven calpain-mediated axonal cytoskeleton damage [68]. Altogether, the ability of P2Y1R to promote astrocyte hyperactivity and consequent glutamate release, to recruit and eventually format microglia response, and to directly increase the susceptibility of neurons to damage, indicate that P2Y1R is endowed with a transcellular capability to catalyze neurodegeneration in different brain disorders (Figure 1), both at the early onset [68,115] and at a chronic stage [44,115,124].

The contribution of the purinergic signaling system to brain pathologies is not limited to P2Y1R. Other P2Rs, adenosine P1Rs, or ectoenzymes involved in the extracellular metabolism of ATP have been associated to the pathogenesis of different brain disorders, displaying both neurodegenerative actions, namely P2X7R, A_2A_R, and CD73 [59,62,64], and neuroprotective actions such as with P2Y2, P2Y4, P2Y12, and P2Y13 receptors (e.g., [63,65,131,132]). Hence, in order to fully comprehend the pathological contribution of P2Y1R to brain disorders and its potential value as a therapeutic target, it is fundamental to contextualize it within the purinergic signaling system. It will be important to understand the hierarchy, cooperation, and/or redundancy between the different elements that comprise the purinergic signaling system and understand how the contribution of purinergic signaling in pathological conditions is orchestrated. Some studies started to shed light on this topic. Besides the contribution of different purinergic receptors to the release of ATP such as P2X7R or A_2A_R [43,51,52,59], microglia P2Y13R prevents astrocyte proliferation induced by P2Y1R [133], and more recently, it was shown that A_2A_R physiologically reduces P2Y1R-driven Ca^2+^ increases in astrocytes, an effect blunted by Aßexposure [134]. This will allow a better comprehension of the contribution of P2Y1R to neurodegeneration, which is fundamental to define an eventual therapeutic strategy targeting P2Y1R, either directly or indirectly, to prevent its deleterious contribution. This may involve a multitarget time-dependent strategy. Since a sustained ATP release and the pathogenic involvement of P2Y1R is an event shared by different acute and chronic brain disorders, such a strategy targeting P2Y1R function may bring a sole therapeutic intervention to the different neurodegenerative disorders. 

## Data Availability

Not applicable.
